# The Role of Cysteine Protease Cathepsins B, H, C, and X/Z in Neurodegenerative Diseases and Cancer †

**DOI:** 10.3390/ijms242115613

**Published:** 2023-10-26

**Authors:** Veronika Stoka, Olga Vasiljeva, Hiroshi Nakanishi, Vito Turk

**Affiliations:** 1 Department of Biochemistry and Molecular and Structural Biology, Jožef Stefan Institute, SI-1000 Ljubljana, Slovenia; olga.vasiljeva@ijs.si; 2 Jožef Stefan International Postgraduate School, SI-1000 Ljubljana, Slovenia; 3 CytomX Therapeutics, Inc., South San Francisco, CA 94080, USA; 4 Department of Pharmacology, Faculty of Pharmacy, Yasuda Women’s University, Hiroshima 731-0153, Japan; nakanishi-h@yasuda-u.ac.jp

**Keywords:** cathepsins, lysosomal exopeptidases, neurodegeneration, Alzheimer’s disease, Parkinson’s disease, Huntington’s disease, amyotrophic lateral sclerosis, multiple sclerosis, neuropsychiatric disorders, cancer

## Abstract

Papain-like cysteine proteases are composed of 11 human cysteine cathepsins, originally located in the lysosomes. They exhibit broad specificity and act as endopeptidases and/or exopeptidases. Among them, only cathepsins B, H, C, and X/Z exhibit exopeptidase activity. Recently, cysteine cathepsins have been found to be present outside the lysosomes and often participate in various pathological processes. Hence, they have been considered key signalling molecules. Their potentially hazardous proteolytic activities are tightly regulated. This review aims to discuss recent advances in understanding the structural aspects of these four cathepsins, mechanisms of their zymogen activation, regulation of their activities, and functional aspects of these enzymes in neurodegeneration and cancer. Neurodegenerative effects have been evaluated, particularly in Alzheimer’s disease, Parkinson’s disease, Huntington’s disease, amyotrophic lateral sclerosis, multiple sclerosis, and neuropsychiatric disorders. Cysteine cathepsins also participate in tumour progression and metastasis through the overexpression and secretion of proteases, which trigger extracellular matrix degradation. To our knowledge, this is the first review to provide an in-depth analysis regarding the roles of cysteine cathepsins B, H, C, and X in neurodegenerative diseases and cancer. Further advances in understanding the functions of cysteine cathepsins in these conditions will result in the development of novel, targeted therapeutic strategies.

## 1. Cysteine Cathepsins: Structural and Functional Aspects

The discovery of lysosomes by Christian de Duve has been crucial for understanding intracellular degradation processes [1]. The release of enzymes from injured lysosomes results in the destruction of their own cells and cell death [2,3].

Lysosomes are present in almost all eukaryotic cells and contain more than 50 acid hydrolases. The primary function of lysosomes, which can degrade and digest proteins, is not to destroy cells but rather to maintain cellular homeostasis and recycle cell constituents, as has been demonstrated in several physiological processes (reviewed in [4,5,6,7,8,9]).

This classical view has changed more recently after the discovery of cysteine cathepsins in the nucleus, mitochondria, cytoplasm, and extracellular space [9,10,11,12,13].

Recent developments in quantitative proteomics and in vivo imaging have elucidated protease specificity profiling and identified physiological substrates [14], resulting from the concept of proteases, including cysteine cathepsins, as degrading enzymes and proteases as key signalling molecules [15,16].

A typical example of signalling is the activation of the pro-apoptotic protein Bid, a member of the Bcl-2 family, which initiates apoptosis [17,18,19,20].

When released from the lysosomes, cathepsins are potentially hazardous and frequently associated with various human pathologies, including cancer [21,22,23,24], cardiovascular diseases [25,26], neurodegeneration [24,27,28], bone disorders and inflammatory diseases [29,30,31], coronavirus disease caused by SARS-CoV-2 [32,33], and, although less investigated, diseases with a genetic deficiency of cysteine cathepsins F, K, C, and H and lysosomal storage diseases [34].

Proteases catalyse irreversible hydrolytic reactions; therefore, their proteolytic activity must be strictly regulated. This can be achieved at multiple levels by various mechanisms, such as gene expression, post-translational modification, autocatalytic activation of their inactive zymogens, or by other proteases targeting specific compartments, intracellular protein processing and degradation, oxidants and endogenous protein inhibitors, or exogenous inhibitors [16,35,36]. Once activated, mature enzymes are proteolytically active and must be regulated by pH, temperature, oxidation, glycosaminoglycans, and endogenous protein inhibitors.

Inhibitors can be classified into emergency and regulatory inhibitors based on their localisation. Emergency inhibitors are normally localised in different cellular compartments than the enzyme, cystatins being a typical example, whereas regulatory inhibitors are often co-localised with their target [16,37,38].

The most well-studied are cystatins (family I25), which are divided into stefins (I25A), cystatins (I25B), and kininogens (I25C) subfamilies [39]. They are competitive, reversible, tight-binding inhibitors that can discriminate between endo- and exopeptidases (more in reviews by [37,38,40,41,42]).

The crystal structure of chicken cystatin has served as the foundation for elucidating the new mechanism of interaction between cystatins and papain-like enzymes [43], which is thus confirmed by the crystal structure of the human stefin B-papain complex [44].

The chicken cystatin nuclear magnetic resonance structure exhibits the same overall fold but also notable differences in some segments of the polypeptide chain that are more similar to those of human stefin B [45].

Recently, thyropins, which are novel protein inhibitors structurally different from cystatins, have been identified. They belong to family I31 of clan IX [39]. The physiologically most important inhibitor of this family is the p41 fragment of the invariant chain, which inhibits several cathepsins and is involved in regulating major histocompatibility complex-II antigen presentation [46,47].

There are numerous synthetic inhibitors, among which epoxysuccinate derivatives were the first identified inhibitors of cysteine cathepsins [48]. One of these, the irreversible inhibitor CA030 (ethyl ester of epoxysuccinyl-Ile-Pro-OH), has been crystallised in complex with cathepsin B. Notably, the Ile-Pro-OH region of CA030 mimics the P1′ and P2′ residues in the substrate. Therefore, this structure initially revealed a substrate-like interaction with the S1′ and S2′ residues of papain-like enzymes [49].

Among lysosomal hydrolases, proteases (also termed peptidases) play an important role. There are 15 cathepsins in humans, which are classified as follows, according to their catalytic type: serine proteases (cathepsins A and G), aspartic proteases (cathepsins D and E), and cysteine proteases (cathepsins B, C, F, H, K, L, O, S, V, W, and X/Z) (Table 1 and Table S1). For consistency, the name cathepsin X has been used throughout the manuscript when referring to cathepsin X or Z, since they refer to the same enzyme that has been simultaneously reported by two independent groups using different names [50,51].

These 11 lysosomal cysteine cathepsins are members of the papain family (C1A) from the cysteine peptidases clan (CA). They are predominantly endopeptidases, except for cathepsins C and X, which are strictly exopeptidases. Moreover, cathepsin B is a carboxydipeptidase, and cathepsin H is an aminopeptidase. Both of these cathepsins are predominantly exopeptidases, exhibiting limited access to their active sites. Cathepsin B contains an insertion of approximately 20 amino acid residues, termed the occluding loop, which blocks the active-site cleft and consecutively removes two amino acids [53]. With increasing pH, the loop becomes flexible, allowing cathepsin B to function as an endopeptidase [54]. An irreversible loss of cathepsin B activity accompanied by structural changes has been observed at neutral or alkaline pH [55]. Meanwhile, the exopeptidase activity of cathepsin B is limited to an acidic pH. Deletion of the occluding loop results in cathepsin B with endopeptidase activity only [56]. Therefore, the pH-dependence of the propeptide binding can be explained by competitive binding of the occluding loop and the propeptides [57]. The crystal structures of the cathepsin B-stefin A complex [58] and the cathepsin B-chagasin complex [59] displace the occluding loop, thus inhibiting the cathepsin B endopeptidase activity.

In vitro studies have demonstrated that only cathepsin L cleaves and activates procathepsin H [60]. Upon activation, the mature enzyme primarily acts as an aminopeptidase, thus cleaving a single N-terminal residue from the polypeptide chain. An octapeptide known as the “mini-chain,” which is disulphide-linked to the main enzyme structure in the narrow active-site cleft in the substrate-binding direction, is responsible for the strong aminopeptidase activity. The mini-chain is positioned in the active-site cleft via carbohydrate residues attached to the enzyme structure [61]. From the 38-residue propeptide, the mini-chain EPQNCSAT octapeptide contains non-primed substrate-binding sites starting at the S2 position. The positioning of the mini-chain and substrate, which is based on the displacement of residues within the active-site cleft, allows cathepsin H to exhibit exopeptidase activity. The crystal structure of stefin A-cathepsin H reveals structural changes along its interaction surface [62]. Recombinant cathepsin H lacking a mini-chain exhibits only endopeptidase activity, confirming that the mini-chain is responsible for the enzyme’s aminopeptidase activity [63].

All amino acid sequences were determined and confirmed by bioinformatics analysis of the human genome draft sequence [64]. While the majority of the above-described cysteine cathepsins are ubiquitously expressed, the other four cathepsins, K, S, V, and W (also named lymphopain), show a more restricted cell- or tissue-specific distribution, suggesting their specific cellular functions [65].

Cysteine cathepsins are optimally active at acidic pH values (pH 3.5–6.0) and in a reducing environment and are mostly unstable and inactivated at neutral pH values, except cathepsin S, which is stable and active at neutral or slightly alkaline pH values [66]. Heparin-like glycosaminoglycans can potentiate the endopeptidase activity of cathepsin B at alkaline pH values by interacting with heparin and heparan sulphate in the occluding loop of the enzyme [67]. Recently, dual activities, namely dipeptidyl carboxypeptidase and endopeptidase activities, of cathepsin B under both acidic and neutral pH conditions have been reported [68]. Furthermore, these researchers also developed a novel synthetic tripeptide substrate that is highly specific for monitoring high cathepsin B activity at acidic to neutral pH values [69].

**Figure 1:**
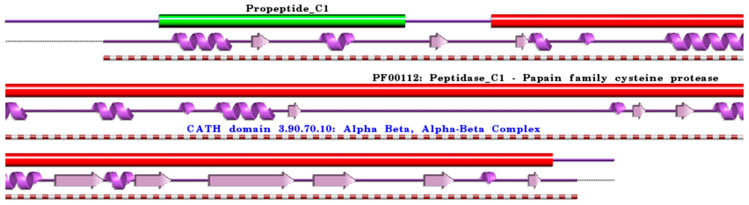
Schematic representation of aligned PDB and UniProt sequences of human procathepsin B. The upper panel shows the domain composition of human procathepsin B [76] (UniProtKB ID P07858) [52], namely the “Activation peptide” (PfamA domain: Propeptide_C1) (green) and the “Cathepsin B chain” (PfamA domain: PF00112: Peptidase_C1) (red). In the lower panels, a schematic “wiring diagram” of human procathepsin B (PDB ID: 3pbh:A, [77]) 2D structure highlights the helices (purple springs) and strands (pink arrows), along with the CATH structural hierarchy classification of protein domain structures, which clusters proteins at four major levels, namely Class (C), Architecture (A), Topology (T), and Homologous superfamily (H). The figure was generated using the PDBsum web server [78]. The sequence alignment is shown in Supplementary Materials (File S1). Details regarding molecular processing are provided in the main text and Table 1.

**Figure 2:**
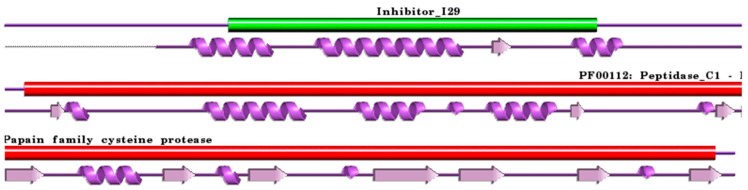
Schematic representation aligned PDB and UniProt sequences of human procathepsin H. The upper panel shows the domain composition of human procathepsin H [79] (UniProtKB ID P09668) [52], namely the “propeptide region” (PfamA domain: Inhibitor_I29) (green) and the “Cathepsin H chain” (PfamA domain: PF00112: Peptidase_C1) (red). In the lower panel, a schematic “wiring diagram” of human procathepsin H (PDB ID: 6czk:A, [60]) 2D structure highlights the helices (purple springs) and strands (pink arrows). The figure was generated using the PDBsum web server [78]. The sequence alignment is shown in Supplementary Materials (File S2). Details regarding molecular processing are provided in the main text and Table 1.

**Figure 3:**
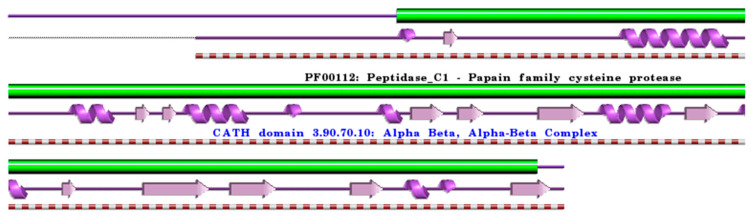
Schematic representation of the aligned PDB and UniProt sequences of human procathepsin X. The upper panel shows the domain composition of human procathepsin X [50,51] (UniProtKB ID Q9UBR2) [52], namely the “Cathepsin X chain” (PfamA domain: PF00112: Peptidase_C1) (green). In the lower panels, a schematic “wiring diagram” of human procathepsin X (PDB ID: 1deu:A, [75]) 2D structure highlights the helices (purple springs) and strands (pink arrows), along with the CATH structural hierarchy classification. The figure was generated using the PDBsum web server [78]. The sequence alignment is shown in Supplementary Materials (File S3). Details regarding molecular processing are provided in the main text and Table 1.

**Figure 4:**
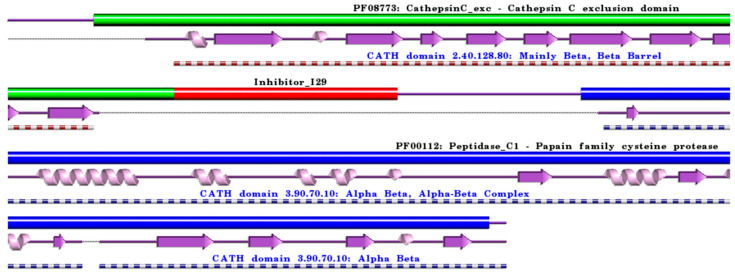
Schematic representation of aligned PDB and UniProt sequences of human procathepsin C. The upper panel highlights the domain composition of human procathepsin C [80] (UniProtKB ID P53634) [52], comprising the mature form of the enzyme, namely the “Cathepsin C exclusion domain chain” (PfamA domain: PF08773: CathepsinC_exc) (green), the “Cathepsin C chain” (PfamA domain: PF00112: Peptidase_C1) (blue), and the “propeptide” (PfamA domain: Inhibitor_I29) (red). In the lower panels, a schematic “wiring diagram” of human cathepsin C (PDB ID: 3pdf:A, [81]) 2D structure highlights the helices (purple springs) and strands (pink arrows), along with the CATH structural hierarchy classification. The figure was generated using the PDBsum web server [78]. The sequence alignment is shown in Supplementary Materials (File S4), where it is evident that the propeptide (Inhibitor_I29) is absent in the 3D structure. Details regarding molecular processing are provided in the main text and Table 1.

Lysosomal cathepsins are synthesised as preproenzymes (Figure 1, Figure 2, Figure 3 and Figure 4). After removal of the N-terminal signal peptide in the endoplasmic reticulum, the resulting inactive proenzymes are transported to late endosomes or lysosomes, where the prodomain (propeptide) is removed by limited proteolytic processing to obtain active mature enzymes (Figure 1, Figure 2, Figure 3 and Figure 4). This activation process occurs autocatalytically at acidic pH values as a combination of unimolecular and bimolecular processes [70]. We further proposed a model for the autocatalytic activation of cysteine cathepsins. This involved the low catalytic activity of procathepsin B in dissociating the propeptide from the active-site cleft as the first unimolecular step during zymogen activation. The second step is the bimolecular proteolytic removal of the propeptide [71]. This activation is facilitated by glycosaminoglycans [72,73]. In contrast, procathepsin C is activated to its mature form by cathepsin L and S but not by autocatalytic processing [74]. Similarly, procathepsin X is incapable of autocatalytic processing but can be processed in vitro using cathepsin L under reducing conditions [75].

The crystal structures of human procathepsins, including those of procathepsin B [77,82], H [60], X [75], and L [83], have revealed that these propeptides share the same fold despite differences in amino acid sequences and lengths (Figure S1).

Most propeptides contain approximately 100 amino acid residues; the shortest propeptide of cathepsin X contains only 38 residues [50,75], whereas the longest are cathepsin C, with 206 amino acid residues [80], and cathepsin F, which has 251 residues and contains a cystatin-like domain unique to cysteine cathepsin zymogens [84]. Propeptides fold on the enzyme surface, covering the catalytic site and acting as inhibitors, suggesting that this mode of inhibition is common to all enzymes of the papain superfamily [83,85]. The propeptides unfold at an acidic pH, thus exposing the active site of the enzyme and suggesting a mechanism of acidic zymogen activation [86].

During activation, propeptides from endopeptidases, including cathepsin B, dissociate from the enzyme surface, whereas exopeptidases, such as cathepsin C and cathepsin H, show different activation processes. The crystal structure of cathepsin C, which is unique among papain-like enzymes, reveals that the mature enzyme is a tetramer composed of four identical papain-like endopeptidases and four exposed active sites [87,88]. The proenzyme is a dimer [74], which oligomerizes into a tetramer. The additional domain, termed the “exclusion” domain, with no homology to papain-like enzymes, contributes to the tetrameric structure and is extended to the active site cleft, thus limiting access to the polypeptide apart from the N-terminus. The crystal structure shows that the mature enzyme contains 119 residues in the exclusion domain (from Asp 1 to 119) and 233 residues in the two papain-like domains (from Leu207 to Leu439). The 87-residue propeptide is cleaved off (from Thr120 to His206) during activation of the proenzyme by cathepsin L and cathepsin S [74]. It blocks not only the active site of the enzyme but also prevents oligomerization [88]. The active site is blocked beyond the S2 binding site by the exclusion domain (more details are provided in [88]). Cathepsin C, also called dipeptidyl peptidase I, sequentially cleaves dipeptides and is the only cathepsin that requires halide ions for its activity. Recombinant cathepsin C, which lacks its exclusion domain, is a monomer with endopeptidase activity [89].

**Figure 5:**
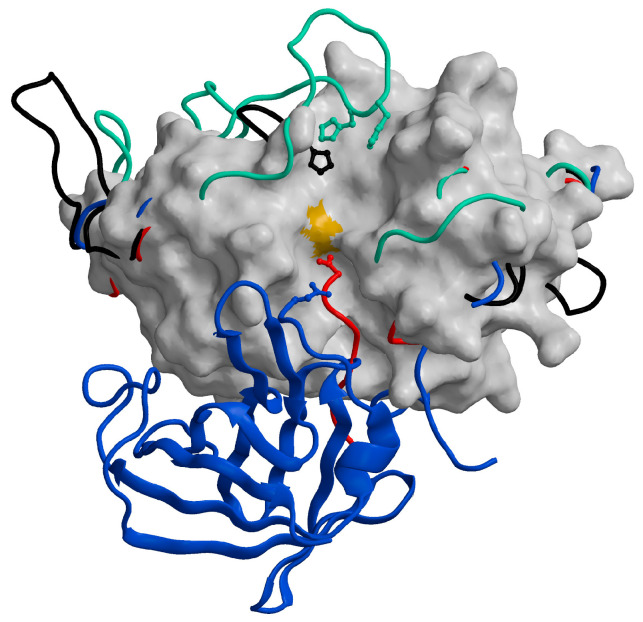
Distinctive features of cysteine cathepsins with exopeptidase activity. The chain traces of the structural elements responsible for the exopeptidase activity of cathepsins B (1huc; [53]; green), C (1k3b; [88]; blue), H (8pch; [61]; red), and X (1ef7; [92]; black) are highlighted over the surface of the endopeptidase cathepsin L (1icf) [94]. The figure has been generated using the MAIN programme [95] and modified from [65].

The mature forms of all cysteine cathepsins share similar sequences and a typical papain-like fold, which consists of two domains forming a “V” active site cleft with a catalytic dyad of Cys25 and His159 on opposite sides of the domains, forming a thiolate-imidazolium ion pair responsible for enzyme activity [90]. All cysteine cathepsins are monomers with molecular weights (MWs) of approximately 30 kDa, with the exception of tetrameric cathepsin C (200 kDa) [87] and the active homodimer of cathepsin X (55 kDa) [91]. From the crystal structures of the exopeptidases cathepsin B [53], cathepsin H [61], cathepsin C [88], and cathepsin X [92], it is evident that their exopeptidase activities result from additional structural elements such as loops (cathepsins B and X) and propeptide regions (cathepsins C and H) (Figure 5). Cathepsin X has later been reported to be a carboxymonopeptidase [93]. Detailed information is available in the original structural papers and reviews [65,90].

Regarding the following sections, to our knowledge, this is the first review to provide an in-depth analysis regarding the roles of cysteine cathepsins B, H, C, and X in neurodegenerative diseases and cancer; notably, these cathepsins exhibit exopeptidase activity. Nevertheless, some recent reviews have focused on some of these cathepsins in either neurodegeneration [24,27,28,96,97,98,99] or cancer [23,24].

## 2. Cathepsins B, H, C, and X in Neurodegenerative and Neuropsychiatric Disorders

Age-related neurodegenerative disorders are often termed ‘proteinopathies’ due to the presence of misfolded and aggregated proteins that lose their physiological roles and acquire neurotoxic properties [100,101]. Notably, most neurodegenerative disorders share an endolysosomal dysfunction due to the accumulation and spread of oligomeric forms of neurotoxic proteins [100,102], where cathepsins play an important role [27,96,97,103,104,105,106,107,108,109]. Several proteins associated with neurodegenerative diseases have been identified as cathepsin substrates [98]. Recently, cysteine cathepsins were also found to be involved in neuroinflammation [24,110,111,112,113,114,115,116,117,118,119,120], a process closely linked to synaptic dysfunction and neurodegeneration [121,122,123]. Neuroinflammatory processes considerably impact the pathology of neuropsychiatric disorders [124]. Therefore, the role of cathepsins in these conditions has attracted increasing interest [125,126]. Noteworthy, cathepsin B [127,128,129,130] and cathepsin X [116,117], which have been linked to neuronal damage under a variety of pathological conditions [128,129,131], are released by activated microglia.

α-Synuclein (α-Syn) aggregation, which is clinically found in the inclusion bodies of post-mortem brain tissues from patients with Parkinson’s disease (PD) [132], activates microglia [133,134,135]. Overall, neuroinflammation in activated microglia is presumably neurotoxic [116,127,135,136,137].

**Figure 6:**
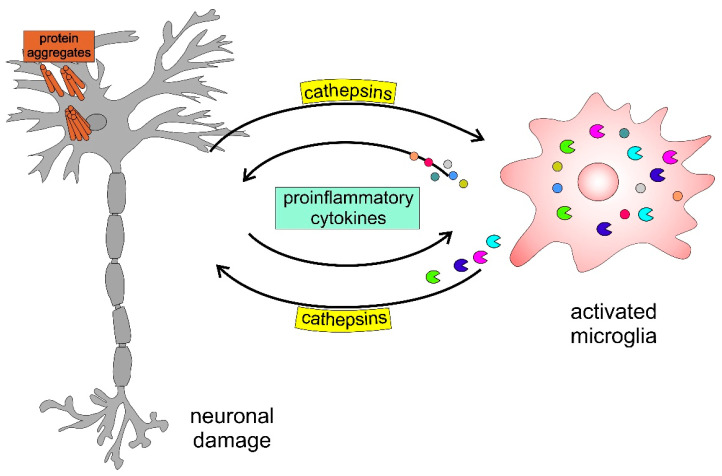
Schematic model of neuronal-microglial crosstalk with emphasis on the role of cathepsins in neurodegenerative diseases.

Figure 6 presents a schematic model highlighting the involvement of cathepsins in neurodegenerative disorders. The accumulation of protein aggregates, such as amyloid-β (Aβ), α-Syn, and mutated huntingtin, activates microglia, inducing the activation and release of cysteine cathepsins (i.e., B, H, C, X) and proinflammatory cytokines, including interleukin-1β (IL-1β) and tumour necrosis factor-α (TNF-α), which further enhance this self-propelling neurotoxicity, leading to neurodegeneration.

### 2.1. Roles of Cathepsins B and X in Alzheimer’s Disease

Alzheimer’s disease (AD) is a progressive neurodegenerative disease frequently associated with memory deficits and cognitive decline [138,139]. Extracellular Aβ and amyloid precursor protein (APP) deposits, intracellular neurofibrillary tangles, dystrophic neuritis, and amyloid angiopathy are the neuropathological markers of AD [140].

The “amyloid cascade hypothesis”, presented by Hardy and Higgins in the 1990s, claims that the pathology of AD is caused by the deposition of Aβ, the primary component of plaques, consequently causing neurofibrillary tangles, cell death, vascular damage, and dementia [141]. Although this concept has influenced and guided much academic and pharmaceutical research, Aβ is necessary but insufficient to cause AD [142].

In contrast, Cataldo and Nixon proposed that APP within senile plaques is processed by lysosomal proteases principally derived from degenerating neurons [143]. Ten years later, Nixon proposed the “protease activation cascade”, which is pertinent to the pathogenesis of sporadic AD and entails the early and progressive activation of proteolytic systems such as the calpain-calpastatin and endolysosomal systems, but not exclusively [105]. Using thioflavin T fluorescence, liquid chromatography, and mass spectrometry, Lambeth and Julian recently investigated the proteolysis of Aβ by cathepsins B, H, L, and D and demonstrated that all Aβ fibril morphologies are resistant to cathepsin digestion [144].

Since the development of therapeutic agents for AD based on the amyloid cascade hypothesis was unsuccessful, considerable attention was given to the “amyloid cascade-inflammatory hypothesis” [145]. AD presumably results from an inflammatory response induced by extracellular Aβ deposits, which are later enhanced by tau aggregates. The inflammatory response driven by activated microglia increases with disease progression [145]. Importantly, cathepsins play a crucial role in the activation of microglia during chronic neuroinflammation [120,130].

On the one hand, cathepsins and other lysosomal hydrolases accumulate within senile plaques in the brains of patients with AD [105,146]. On the other hand, cathepsins are involved in the initiation and mediation of apoptosis and other forms of cell death [128]. Thus, dysfunction of the lysosomal system is a potential pathogenic mechanism in AD-related neurodegeneration. Cathepsin X is also associated with plaques in patients with AD [147] and AD transgenic mouse models APP/PS1 [147] and Tg2576 [148].

Moreover, Sun et al. proposed the “cystatin C-cathepsin B axis” and showed that cystatin C regulates soluble Aβ and Aβ-associated neuronal deficits by inhibiting cathepsin B-induced Aβ degradation [149]. Bernstein and Keilhoff recently reviewed the putative roles of cathepsin B in AD pathology and highlighted that it shows a neuroprotective effect by lowering Aβ levels and improving neuronal dysfunction; in contrast, it may also contribute to AD pathology by acting as a β-secretase and generating pyroglutamate Aβ [150].

Recently, Nixon proposed a multifactorial disease model wherein β-amyloidogenesis and the endolysosomal network have been identified as essential for the cause and progression of AD [151].

Bai et al. showed that oxidative stress activates the NLRP3 inflammasome by upregulating cathepsin B activity, thus highlighting the role of cathepsin B in neuroinflammation and as a potential target in AD therapy [152]. Recently, Nakanishi reviewed how intracellular and extracellular proteolytic mechanisms of microglial cathepsin B contribute to inflammatory brain disorders and brain aging [130]. Nuclear factor-κB (NF-κB) is activated by proteolytic degradation of κBα inhibitor (IκBα), an endogenous inhibitor of NF-κB, and subsequent nuclear translocation of NF-κB. The signalling-induced degradation of IκBα is mediated by the ubiquitin-proteasome system [153]. However, autophagy machinery may also be involved in IκBα degradation [154]. In activated microglia, cathepsin B induces the autophagic degradation of IκBα, leading to chronic neuroinflammation [155]. Various studies have demonstrated the involvement of microglial cathepsin B in cell death and Aβ clearance [128,129,130]. Therefore, the phagocytic clearance of Aβ by microglia may potentially resolve chronic neuroinflammation in AD [130]. Recently, Ni and Wu reviewed the molecular mechanisms governing the crosstalk between systemic inflammation and neuroinflammation. They suggested that disseminating inflammation indicates a negative spiral between systemic diseases and AD and proposed that inhibition of cathepsin B or S may delay the onset of AD and enable early intervention [155]. A systematic review of human post-mortem immunohistochemical studies and bioinformatics analyses revealed the complexity of AD reactive astrogliosis, which involves cathepsins [156]. In addition, Thygesen et al. demonstrated the involvement of cathepsin X expressed in myeloid cells of the central nervous system (CNS) in AD [157].

Since compromised synapses and cognition are improved by safely increasing protein clearance through modulated cathepsin B, Hwang et al.’s research supports the idea that early cathepsin B upregulation is a disease-modifying therapy that may also retard the progression of mild cognitive impairment to dementia [158].

The neuropathology of AD, traumatic brain injury, and other related brain disorders have all been linked to cathepsin B, according to extensive research from Hook’s lab [96,159,160]. Cathepsin B is possibly redistributed from the lysosomes to the cytosol, where it initiates cell death and inflammatory processes linked to neurodegeneration [96]. Therefore, cathepsin B has been proposed as a potential target for AD prevention and therapy [161,162,163,164].

Dunlop and Carney reported that L-serine selectively induces the activity of autophagic-lysosomal enzymes, cathepsins B and L, but not proteasome-hydrolysing activities, thus contributing to its neuroprotective effect [164]. Moreover, Cecarini et al. demonstrated that metabolites such as phenyl-γ-valerolactones exert neuroprotective activity by regulating intracellular proteolysis and confirmed the role of cathepsin B in autophagy [162]. However, the repertoire of potent small molecules that act as potential cathepsin B inhibitors is expanding. This includes E64d [161], pyridine, acetamide, and benzohydrazide compounds [163], and various natural and synthetic heterocyclic scaffolds [165].

Very recently, Cheng et al. reviewed the use of nanomedicines targeting AD lesions as a more suitable strategy than conventional therapy for AD treatment ([166] and references therein).

### 2.2. Roles of Cathepsins B and X in Parkinson’s Disease

Parkinson’s disease (PD) is the second most common age-associated neurodegenerative disorder and is characterised by the loss of dopaminergic neurons and the presence of α-Syn-containing aggregates in the substantia nigra pars compacta. Chronic neuroinflammation is a hallmark of PD pathophysiology [167], and microglial cathepsin B has been proposed as a key driver of inflammatory brain diseases and brain ageing [130].

To investigate the mechanisms underlying astrocyte ATP13A2-regulated lysosomal function and neuroinflammation following 1-methyl-4-phenylpyridinium treatment, Qiao et al. used a PD model of cultured primary neurons and astrocytes from the mouse midbrain [168]. The authors showed that the lack of ATP13A2 increases lysosomal membrane permeabilization and cathepsin B release, which in turn exacerbates activation of the NLRP3 inflammasome to produce excess IL-1β from astrocytes, thus suggesting a direct link between astrocyte lysosomes and neuroinflammation [168].

Codolo et al. demonstrated that although the monomeric and fibrillar α-Syn forms can promote pro-IL-1β expression, following the engagement of Toll-like receptor (TLR) 2, secretion of the mature cytokine is specific to the fibrillated protein, a process involving NLRP3 inflammasome activation [169]. This relies on the phagocytosis of fibrillar α-Syn, followed by the increased production of reactive oxygen species (ROS) and the release of cathepsin B into the cytosol [169]. In addition, Freeman et al. reported that α-Syn aggregates can induce lysosome rupture following endocytosis in neuronal cell lines via a mechanism that induces a cathepsin B-dependent ROS increase in target cells [170]. They also observed that α-Syn aggregates induce inflammasome activation in THP-1 cells [170]. NLRP3 inflammasome activation by α-Syn upon microglial endocytosis and subsequent lysosomal cathepsin B release has also been confirmed in the midbrain of PD model mice and in the serum of patients with PD [171]. Thus, fibrillar α-Syn released during neuronal degeneration endogenously triggers the cathepsin B-mediated inflammatory response in PD, which likely precedes neurodegeneration [170,171].

In contrast, cysteine cathepsin activity is essential for the lysosomal degradation of α-Syn [172] and C-terminal α-Syn truncations in PD [173]. Hu et al. showed that α-Syn is primarily degraded in the lysosomes, whereas the Leucine-rich repeat serine/threonine-protein kinase 2 (LRRK2) G2019S mutation, which is the most common genetic cause of PD, inhibits α-Syn degradation and promotes its aggregation. Moreover, LRRK2 G2019S decreases the activity of lysosomal enzymes, including cathepsins B and L, indicating that the inhibitory effect of LRRK2 G2019S on α-Syn degradation could underlie the pathogenesis of aberrant α-Syn aggregation in PD with LRRK2 mutation [174]. In addition, α-Syn fibril-induced intracellular aggregate formation requires lysosomal function, which is dependent on cathepsin B and not aspartic cathepsin D [175].

Recently, Blauwendraat et al. demonstrated a decrease in active cathepsin B protein levels in iPSC-derived neurones among glucosylceramidase β1 (GBA) variant carriers compared to those in non-carriers, suggesting a further reduction in lysosomal protease function in these cases. Moreover, α-Syn levels remain unaltered in the forebrain neurones carrying the GBA variant, suggesting that the overall reduction in lysosomal proteases allows for a faster accumulation of α-Syn aggregates as neurones age [176].

In contrast, Nelson et al. revealed that cathepsin D activity significantly decreases in the temporal cortex of patients with late-stage PD in the absence of cathepsin B as well as glucocerebrosidase (GCase) activity [177]. Moreover, a significant correlation exists between a decrease in GCase activity and an increase in p129S-α-Syn, whereas cathepsin D or cathepsin B do not correlate significantly with α-Gal A activity or levels [177].

More recently, Kim et al. demonstrated that ceramide activates cathepsin B and identified a novel role for cathepsin B in mediating prosaposin cleavage to form saposin C, the lysosomal coactivator of GCase [178]. Senkevich et al. reported that genetic modifiers such as LRRK2, endosomal/lysosomal proton channel TMEM175, α-Syn→(SNCA), and cathepsin B (*CTSB*) can either affect GCase activity or modulate the risk and age at the onset of GBA-associated PD [179]. In addition, Kim et al. suggested that loss of GBA1, Sphingomyelin phosphodiesterase (SMPD1), or Galactocerebrosidase (GALC) function in PD causes lysosomal ceramide deficiency; reduced ceramide-mediated cathepsin B activation in the lysosomes subsequently impairs the processing of prosaposin to saposin C, ultimately impairing GCase activity [178]. Kim et al. were the first to report a mechanistic link between ceramide and cathepsin B in regulating GCase activity and suggested that targeting lysosomal ceramide or cathepsin B is an important therapeutic strategy for activating GCase in PD and related disorders [178].

Recently, Pišlar et al. reported the upregulation of cathepsin X in the 6-hydroxydopamine (6-OHDA) model of PD and suggested cathepsin X as an important factor leading to the progressive loss of dopaminergic neurones and a potential therapeutic target for PD intervention [108]. Moreover, dopamine neuron cell death on treatment with 6-OHDA induces the loss of tyrosine hydroxylase, caspase-3 activation, intracellular ROS generation, and mitochondrial dysfunction, including the release of cytochrome c and an imbalanced Bax/Bcl-2 ratio [109]. This process is prevented by the cathepsin X inhibitor AMS36, which interferes with NF-κB activation by blocking IκBα degradation and preventing NF-κB nuclear translocation [109]. In addition, Lee et al. showed that PC12 cells exposed to 6-OHDA exhibit lysosomal dysregulation, caspase activation, and cell death, which are attenuated by the inhibitors pepstatin A and DEVD-Cho, whereas the cathepsin B inhibitor, CA-074Me, fails to protect cells [180]. In contrast, Wu et al. reported that the autophagy/lysosomal pathway is involved in the 6-OHDA-induced death of PC12 cells. The authors showed that overactive autophagy due to mitochondrial disability increases cathepsin B expression and diminishes Bcl-2 expression, whereas necrostatin-1 exerts a protective effect against injury in dopaminergic neurones [181].

Recently, Milanowski et al. identified *CTSB* p.Gly284Val as a rare variant in PD pathogenesis, suggesting that the *CTSB* locus harbours variants with varying penetrance that determine disease risk [182]. This finding expands the known repertoire of PD-linked genes (PARK1-21) [97,183,184] and the comprehensive genetic database for PD (Gene4PD) [185].

### 2.3. Roles of Cathepsins B, H, and X in Huntington’s Disease

Huntington’s disease (HD) is a progressive, fatal, autosomal dominant neurodegenerative disorder characterised by uncontrolled excessive motor movements and cognitive and emotional deficits [186,187,188,189].

Early studies by Mantle et al. reported a significant increase in protease activity, particularly of cathepsins H and D, in the brain tissue of patients with HD [104]. Nagata et al. provided direct evidence of abnormalities in HD tissues outside the brain under basal conditions by examining patient lymphoblasts. The authors reported pronounced vacuole formation with huntingtin remnants and cathepsin B staining, suggesting autophagy [190]. Later, Zhang et al. used an HD mouse model to demonstrate involvement of the p53 pathway in signalling, both autophagy and apoptosis, a process involving active cathepsins B and D [191].

Moreover, Kegel et al. identified the endolysosomal pathway as the main pathway for the removal of excess huntingtin, and lysosomal activity may regulate the cleavage of N-terminal fragments, which later aggregate in the nuclear and cytoplasmic inclusions of HD neurones [192]. Several proteases, including cathepsins B, L, X, and D, caspases, calpain, metalloproteases, and proteasomes, contribute to the N-terminal proteolysis of mutant huntingtin [107,192,193,194,195,196].

Interestingly, using cathepsin-deficient cells and pharmacological inhibitors, cathepsins L and X were found to degrade polyQ proteins and peptides but not other aggregation-prone proteins, suggesting that they may have a crucial role in host defence against the toxic accumulation of polyQ proteins [197]. Lai et al. reported that *scyllo*-inositol promotes the robust degradation of mutant huntingtin protein mediated by lysosomes and proteasomes but not autophagosomes. The rescue of degradation pathways is due to a reduction in mutant polyQ-huntingtin protein levels and is not a direct result of the compound on the lysosome or proteasome [198].

### 2.4. Roles of Cathepsins B, H, and X in Amyotrophic Lateral Sclerosis

Amyotrophic lateral sclerosis (ALS) is a degenerative motor neuron disease with a complex aetiology involving protein misfolding. This feature is shared by other neurodegenerative diseases, although there is a distinct common thread among ALS genes, associating them with the autophagy cascade [199].

To clarify the possible association of ALS neurodegeneration with the endolysosomal system, Kikuchi et al. examined the pathological expression of cysteine cathepsins B, H, and L and aspartic cathepsin D in the anterior horns of 15 ALS cases and five controls [106]. Consequently, only cathepsin B expression was upregulated, suggesting that it may play an important role in motor neuron degeneration in ALS [106]. Recently, Mori et al. showed that autophagy is a common degradation pathway for Bunina Bodies and TAR DNA-binding protein 43 (TDP-43) inclusions, which may explain the frequent coexistence of these inclusions in anterior horn cells in sporadic ALS [200].

Lee et al. demonstrated that proteasome inhibitors, but not cathepsin B inhibitors, increase superoxide dismutase [Cu-Zn] (SOD1) aggregate formation but do not promote cell death, indicating the absence of an association between SOD1 aggregates and cell death in familial ALS [201].

cDNA microarray analysis of post-mortem spinal cord specimens from four patients with sporadic ALS compared to four age-matched non-neurological controls revealed 60 differentially expressed genes, including an increase in the expressions of cathepsins B and D, apolipoprotein E, epidermal growth factor receptor, ferritin, and lysosomal trafficking regulator [202]. Since the findings from patients with sporadic ALS corroborate those of the SOD1 transgenic mouse model, the examined genes are suggested to play a specific role in the pathogenesis of ALS [202].

In addition, Boutahar et al. evaluated the effect of oxidative or excitotoxic stress on the transcriptional profile of ALS-linked mutant SOD1-cultured neurones and observed that both the ubiquitin-proteasome and endolysosomal systems are upregulated in transgenic neuron cultures [203]. Moreover, a meta-analysis of gene expression profiling in ALS consistently confirmed that the differential expressions of cathepsins B and D, GFAP, and SERPINA3 are significant in both the mouse model and patients with ALS [204].

Fukada et al. analysed gene expressions in the spinal cord of SOD1 (L126delTT) Tg mice using a cDNA microarray and identified four genes (*Crym*, *Hspb1*/*Hsp27*, *CtsH*, and *Paip1*) potentially related to the pathogenesis of familial ALS, including the progression of reactive astrocytes and the inflammatory response of microglial cells. In particular, cathepsin H was present in reactive astrocytes and microglial cells, suggesting that its overexpression might be associated with a reaction against misfolded proteins due to failure of the ubiquitin–proteasome system [205].

Gene profiling of skeletal muscles in an ALS mouse model showed that before the onset of overt clinical symptoms and motor neuron death, early changes affect genes involved in detoxification, regeneration, tissue degradation, and cell death. Notably, cathepsin X, metallothionein-1 and -2, ATF3, and galectin-3 genes appear to be regulated in both the skeletal muscle and spinal motor neurons of paralysed ALS mice [206].

In addition, Wendt et al. showed that cathepsin X is critical in degenerative processes during normal aging and under pathological conditions, as it has been found to be upregulated in numerous glial cells in the degenerating brain regions of a transgenic ALS mouse model [147].

Moreover, a neurodegeneration-specific gene expression signature of acutely isolated microglia from an ALS mouse model has revealed co-regulated genes in the lysosome pathway, which include several cathepsins (A, B, D, L, S, X, and E), a host of lysosome enzymes (HexA), membrane markers (Cd68, Cd63, and Lamp1), and components of lysosomal ATPase (Atp6v0d1) [207]. Therefore, cathepsins may be involved in the removal of mutant SOD1 aggregates and neuronal debris in ALS mice [207]. Conversely, Ulbrich et al. reported evidence for a reciprocal influence of SOD1 and stefin B/cystatin B genes and a direct interaction between the two proteins [208].

Watanabe et al. demonstrated that cystatin C, the main component of Bunina bodies in ALS, is an endogenous neuroprotective factor that functions via the coordinated activation of two distinct neuroprotective pathways, namely, induction of autophagy and inhibition of aberrant cathepsin B activity [209].

### 2.5. Roles of Cathepsins B, H, C, and X in Multiple Sclerosis

Multiple sclerosis (MS) affects the CNS and is characterised by inflammation, demyelination, and neurodegeneration [210,211]. Increased cathepsin B levels have been reported in monocytes and macrophages, cells known to be activated in the peripheral blood of patients with MS and implicated as effectors of demyelination [212].

Moreover, biochemical analysis of MS brain tissue suggests that monocytes, macrophages, and reactive astrocytes are potential sources of increased cathepsin B levels [213]. Since proteasomal dysfunction is observed in the white and grey matter of patients with MS, an increase in cathepsin B activity may represent a compensatory mechanism for intracellular protein degradation [214].

To identify the proteases involved in MS pathogenesis, cDNA microarray analysis was performed on the brains of transgenic (*plp^tg^*^/−^) mice, an animal model that closely mimics the failure of remyelination in MS [215]. Cathepsins B, H, and L are upregulated in the microglia and macrophages of the brain white matter, whereas elevated cystatin C expression is found in astrocytes, suggesting that the imbalance between cathepsins and their inhibitors may be cytotoxic to neurones (axons) and oligodendrocytes [215].

Using an animal model of MS, Allan and Yates demonstrated that cathepsin L^−/−^ attenuates myelin oligodendrocyte glycoprotein (MOG) antigen presentation and the development of experimental autoimmune encephalomyelitis (EAE) [216]. In contrast, neither cathepsin B^−/−^ nor cathepsin S^−/−^ showed any effect, whereas their double-mutant mice showed attenuated MOG antigen presentation and EAE development [216]. Moreover, Okada et al. showed that cathepsin H deficiency impairs the TLR3-mediated activation of the interferon regulatory factor 3 (IRF3) and interferon-β (IFN-β) secretion from dendritic cells, thus enhancing Th1 cell differentiation and resulting in early-onset EAE in an animal MS model [217]. Therefore, functional redundancy among cathepsins B, L, and S in EAE suggests that the inhibition of multiple cysteine cathepsins may improve autoimmune disorders, such as MS. In contrast, the inhibition of cathepsin H may have an adverse effect on MS.

Recently, Liang et al. demonstrated that the absence of the cystatin F gene and the resulting disinhibition of cathepsin C aggravate demyelination. This finding may be related to increased expression of the glia-derived chemokine CXCL2, which may attract inflammatory cells to sites of myelin sheath damage, an effect that is reversed by knockdown of the cathepsin C gene [113]. Shimizu et al. showed that the balance between cathepsin C and cystatin F controls remyelination in the brain of *Plp1*-overexpressing mice, a model of chronic demyelinating disease [218]. From the same group, Durose et al. confirmed that cathepsin C and cystatin F are strongly associated with inflammatory demyelination; they demonstrated that the severity of EAE is reduced in the absence of cathepsin C. In contrast, increased microglial cathepsin C expression enhances clinical severity, suggesting that the interaction between cathepsin C and cystatin F plays an essential role in the pathogenesis of inflammatory demyelination in EAE [219].

In addition, cathepsin X propagates IL-1β-driven neuroinflammation, thus providing mechanistic support for the epigenetic risk factors in MS [114]. Haves-Zburof et al. evaluated whether the expression levels of cathepsins B and S and their inhibitors, cystatins B and C, are affected by the MS disease state and therapies (IFN-β and methylprednisolone) and whether they are associated with the IFN-β response phenotype. The authors demonstrated that cathepsin S expression levels are aberrantly elevated in patients with MS, in contrast to cathepsin B. However, the value of cathepsin S and cystatin C as predictive biomarkers for disease type, response to therapy, and the development of new targeted therapies for immune-mediated disorders, such as MS, requires further validation [220].

### 2.6. Roles of Cathepsins B and C in Neuropsychiatric Disorders

Transcriptome analysis of inbred mouse lines selected for low or high anxiety-related behaviour with depression-like behaviour revealed that cathepsin B is responsible for low anxiety in female mice [221]. Assessment of anxiety-related and depression-like behaviours in cathepsin B-deficient mice revealed an increase in depression-like behaviours in females. In contrast, cathepsin C aggravates neuroinflammation involved in behavioural and neurochemical disturbances in acute and chronic stress-induced murine models of depression [222]. In contrast, cathepsin C knockdown partially prevents inflammation, which may help alleviate the symptoms of depression in mice.

The “monoamine hypothesis of depression” postulates that the underlying pathophysiologic basis of depression is the decreased levels of 5-hydroxytriotamin, noradrenalin, and/or dopamine in the CNS. More recently, the “neuroplasticity hypothesis of depression” identified dysfunctional neural plasticity as the pathophysiological basis of depression [223]. The role of cathepsin C in promoting anxiety- and depression-like behaviours may be due to the involvement of cathepsin C in neuroinflammation induced by activated microglia [111,119], because depression-like behaviour induced by cathepsin C overexpression is associated with increased neuroinflammation and decreased 5-hydroxytryptamine levels [222]. In contrast, cathepsin B induces neuroinflammation by activated microglia [120,130] and protects against anxiety- and depressive-like behaviours. Therefore, the mechanisms underlying the protective effect of cathepsin B against these disorders may stem from its role in activity-dependent neuronal plasticity by activating matrix metalloprotease-9 [224,225]. Nevertheless, the specific pathophysiological roles of cathepsins in neuropsychiatric disorders should be elucidated in future studies.

## 3. Cathepsins B, H, C, and X in Cancer

Cysteine proteases play prominent roles in multiple molecular pathways involved in tumour progression and metastasis [21,24,226]. Cathepsin B, the most abundant and ubiquitously expressed exopeptidase of the papain family, is associated with tumour progression in numerous cancer types, including colorectal, breast, lung, pancreatic, and gastric cancer [227,228,229,230,231,232,233]. Cathepsin B expression correlates with increased malignancy and poor prognosis; thus, it has been proposed as a predictive biomarker for oral squamous cell carcinoma [234], cervical cancer [235], endometrial cancer [236], and colorectal cancer [232]. Another carboxypeptidase of the clan CA/C1 cysteine protease family, cathepsin X, has been primarily implicated in the development of gastrointestinal cancers, including colorectal [237,238], gastric [239], liver [240], and pancreatic cancers [241].

Moreover, Wang et al. demonstrated the involvement of cathepsin X in regulating the epithelial-to-mesenchymal transition and invasion in hepatocellular carcinoma [240]. Aminopeptidases H and C are overexpressed in various cancers and are involved in malignant transformations [242,243,244,245]. Cathepsin H regulates the processing of talin, a large focal adhesion protein, thereby promoting PC3 prostate cancer cell progression by modulating integrin activation and adhesion strength [246].

Endopeptidase cathepsin L is implicated in tumourigenesis and the malignant progression of different tumour types [24,247,248]. It may act as a tumour promoter by interfering with the tumour suppressor CDK2-AP1, leading to the progression of breast cancer [249]. Tumour-secreted cytokines, which are closely associated with malignant progression, significantly enhance the transcriptional upregulation of cathepsin L. Moreover, increased promoter activity and cathepsin L synthesis by vascular endothelial growth factor A (VEGF) have been demonstrated in glioblastoma cells [250].

Although lysosomal cysteine cathepsins are predominantly intracellular, they are secreted into the extracellular space under multiple physiological and pathological conditions [10,21]. The secretion of cysteine cathepsins is often accompanied by acidification of the extracellular milieu [251], which is a characteristic feature of the tumour microenvironment (TME). Notably, the slightly acidic pH of tumours provides a favourable environment for extracellular cathepsin activity, thereby promoting the execution of their function.

**Figure 7:**
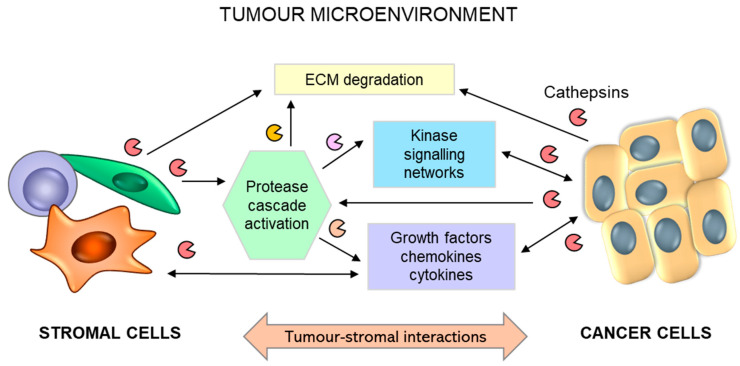
Schematic representation for the role of reciprocal interactions between tumour and stromal cells in promoting tumour progression. Tumour-stromal crosstalk leads to activation of the stroma and overexpression and secretion of proteolytic enzymes, including cathepsins (B, H, C, and X), triggering extracellular matrix (ECM) degradation and the release of soluble factors. Activated stromal cells (macrophages, fibroblasts, and mast cells) secrete additional growth factors, cytokines, and chemokines, which regulate numerous interrelated events leading to tumour progression and metastasis.

In addition to tumour cells secreting substantial levels of cathepsins, tumour stromal cells, such as endothelial cells, mast cells, tumour-associated macrophages, and fibroblasts, are important contributors to the increased levels of cysteine cathepsins in the TME [11,252] (Figure 7). The bulk of cathepsin B and X activity in several cancer types emanates from immune cells of the myeloid lineage [253], such as peritumoral macrophages [254,255,256,257] and myeloid-derived suppressor cells [258]. Secreted cysteine cathepsins can participate in the degradation of extracellular matrix (ECM) proteins, such as E-cadherin [259], collagen IV [260,261], or tenascin-C [262].

Nevertheless, more specific roles of cysteine cathepsins have recently been discovered in modulating extra- and intracellular signal transduction pathways, which can also be executed through the shedding of receptors and adhesion molecules or the processing of respective cytokines and growth factors [11]. It was recently demonstrated that cathepsin C can promote proliferation and metastasis in hepatocellular carcinoma through activation of the TNF-α/MAPK (p38) signalling pathway [263]. Moreover, cathepsin B expression is implicated in regulating TGF-β1 signalling [264] and MAP and PI3 kinase pathways in malignant meningiomas [265].

Genetically engineered mouse models, in combination with genetic ablation or the overexpression of specific proteases, are valuable research tools for elucidating the multiple roles of cathepsins in tumorigenesis and cancer progression. Critical roles of cathepsins B and X in the carcinogenesis, progression, and metastasis of breast cancer have been discovered using a transgenic MMTV-PymT model of metastasising breast cancer [254,257,266]. Furthermore, the impact of cathepsin B on tumour formation and progression has been confirmed in multiple models, including the pancreatic cancer RIP1-Tag2 model [259] and the renal cell carcinoma xenograft model [267]. Although no data are available on the role of cathepsin H in MMTV-PymT breast cancer progression, cathepsin H depletion significantly impairs the establishment and maintenance of tumour vasculature and reduces the tumour burden in the RIP1-Tag2 model of pancreatic islet carcinogenesis [268]. Notably, a cathepsin C tumour-promoting effect has been demonstrated in a squamous cell carcinoma K14-HPV16 model [269], but not in RIP1-Tag2 [259] or MMTV-PymT [269] transgenic mouse models. In summary, the functions of individual proteases may be hardwired into a specific tissue paradigm and thus depend on the cancer type and biology of the primary and metastatic lesion host tissues.

Cathepsin activity exists within a larger integrated network of protease activities known as the protease web [270]. Through interactions with other proteases and their inhibitors, cathepsins can alter the general proteolytic activity within the TME. In addition to directly regulating multiple processes involved in tumour progression and metastasis, many proteases can indirectly impact the activation of multiple cascades of enzymatic activities [271,272]. This can be illustrated by cathepsin B processing of the urokinase-type plasminogen activator (pro-uPA) pro-form, thus converting plasminogen into plasmin [273], which may activate zymogens of matrix metalloproteinases, and thus, together with the precursor proteases of this proteolytic activation cascade, execute the numerous functions associated with tumour progression and metastasis [274]. Notably, these proteolytic webs or networks can interact with other important signalling pathways in tumour biology, including cytokines, chemokines, and kinases [275].

In summary, as essential elements of the proteolytic network balance, cysteine cathepsins B, X, C, and H are involved in multiple steps of cancer development and progression. Therefore, elucidating their roles in tumour biology and regulating relevant signalling pathways can be utilised as novel targeted anticancer therapeutic approaches.

## 4. Conclusions

Since the discovery of lysosomes and lysosomal cathepsins, the understanding of intracellular proteolysis and its role in normal biology and disease states has advanced rapidly. Major progress in this area particularly occurred with the determination of the crystal structures of cathepsins, including B, H, and C, and the last discovered cathepsin, X. Endogenous protein inhibitors and synthetic inhibitors are crucial for understanding the mechanism of interaction with their target enzymes and structure-function relationships, both of which are of crucial importance for the treatment of various diseases, including neurodegeneration and cancer. Currently, research is focused on channelling the existing knowledge to detect proteases in various diseases and treat their overexpression. Activity-based probes are under development, but the identification of physiological substrates remains unexplored, which is expected to be addressed using mass spectroscopy. The finding that cathepsins act as signalling molecules requires an understanding of their signalling pathways and their regulation. Therefore, further basic research is required to develop new therapeutic approaches in the near future. Advances in understanding the function of cysteine cathepsins in neurodegeneration, cancer, and other diseases will result in the development of novel, targeted therapeutic strategies.

## Figures and Tables

**Table 1:** Background information on all 15 human cathepsins (11 cysteine, 2 serine, and 2 aspartic proteases).

Family	Protein Name	UniProtKB Entry ID	Gene Name	Protein Processing	Length (AA)
Peptidase C1	Cathepsin B	P07858	*CTSB*	SIGNAL 1..17; PROPEP 18..79 “Activation peptide”; PROPEP 334..339; CHAIN 80..333 “Cathepsin B”; CHAIN 80..126 “Cathepsin B light chain”; CHAIN 129..333 “Cathepsin B heavy chain”	339
Peptidase C1	Dipeptidyl peptidase I (Cathepsin C)	P53634	*CTSC*	SIGNAL 1..24; PROPEP 135..230; CHAIN 25..134 “Dipeptidyl peptidase 1 exclusion domain chain”; CHAIN 231..394 “Dipeptidyl peptidase 1 heavy chain”; CHAIN 395..463 “Dipeptidyl peptidase 1 light chain”	463
Peptidase C1	Cathepsin F	Q9UBX1	*CTSF*	SIGNAL 1..19; PROPEP 20..270 “Activation peptide”; CHAIN 271..484 “Cathepsin F”	484
Peptidase C1	Cathepsin H	P09668	*CTSH*	SIGNAL 1..22; PROPEP 23..97 “Activation peptide”; PROPEP 106..115; PEPTIDE 98..105 “Cathepsin H mini chain”; CHAIN 116..335 “Cathepsin H”; CHAIN 116..292 “Cathepsin H heavy chain”; CHAIN 293..335 “Cathepsin H light chain”	335
Peptidase C1	Cathepsin K	P43235	*CTSK*	SIGNAL 1..15; PROPEP 16..114 “Activation peptide”; CHAIN 115..329 “Cathepsin K”	329
Peptidase C1	Cathepsin L (Cathepsin L1)	P07711	*CTSL*	SIGNAL 1..17; PROPEP 18..113 “Activation peptide”; PROPEP 289..291; CHAIN 114..333 “Cathepsin L”; CHAIN 114..288 “Cathepsin L heavy chain”; CHAIN 292..333 “Cathepsin L light chain”	333
Peptidase C1	Cathepsin O	P43234	*CTSO*	SIGNAL 1..23; PROPEP 24..107 “Activation peptide”; CHAIN 108..321 “Cathepsin O”	321
Peptidase C1	Cathepsin S	P25774	*CTSS*	SIGNAL 1..16; PROPEP 17..114 “Activation peptide”; CHAIN 115..331 “Cathepsin S”	331
Peptidase C1	Cathepsin L2 (Cathepsin V)	O60911	*CTSV*	SIGNAL 1..17; PROPEP 18..113 “Activation peptide”; CHAIN 114..334 “Cathepsin L2”	334
Peptidase C1	Cathepsin W	P56202	*CTSW*	SIGNAL 1..21; PROPEP 22..127; CHAIN 128..376 “Cathepsin W”	376
Peptidase C1	Cathepsin Z (Cathepsin X)	Q9UBR2	*CTSZ*	SIGNAL 1..23; PROPEP 24..61 “Activation peptide”; CHAIN 62..303 “Cathepsin Z”	303
Peptidase S10	Lysosomal protective protein (Cathepsin A)	P10619	*CTSA*	SIGNAL 1..28; CHAIN 29..480 “Lysosomal protective protein”; CHAIN 29..326 “Lysosomal protective protein 32 kDa chain”; CHAIN 327..480 “Lysosomal protective protein 20 kDa chain”	480
Peptidase S1	Cathepsin G	P08311	*CTSG*	SIGNAL 1..18; PROPEP 19..20 “Activation peptide”; PROPEP 245..255; CHAIN 21..244 “Cathepsin G”; CHAIN 21..243 “Cathepsin G, C-terminal truncated form”	255
Peptidase A1	Cathepsin D	P07339	*CTSD*	SIGNAL 1..20; PROPEP 21..64 “Activation peptide”; CHAIN 65..412; “Cathepsin D”; CHAIN 65..162 “Cathepsin D light chain”; CHAIN 169..412 “Cathepsin D heavy chain”	412
Peptidase A1	Cathepsin E	P14091	*CTSE*	SIGNAL 1..19; PROPEP 20..53 “Activation peptide”; CHAIN 54..396 “Cathepsin E form I”; CHAIN 57..396 “Cathepsin E form II”	396

The data was retrieved from the UniProtKB database [52]. An alternative protein name is indicated in parenthesis. The protein’s length is indicated by its amino acid residue number (AA). The domain boundaries upon protein processing for the signal peptide (SIGNAL), propeptide (PROPEP), and mature form (CHAIN) are indicated accordingly.

## Data Availability

The data presented in this review article were obtained from published articles and publicly accessible databases. All sources and references were appropriately cited within the article. All data are provided within the manuscript and the Supplementary Materials accordingly.

## Supplementary Materials

The following supporting information can be downloaded at: https://www.mdpi.com/article/10.3390/ijms242115613/s1, Table S1: Classification of human cathepsins according to the MEROPS database of proteolytic enzymes; File S1: Sequence alignment of the UniProt and PDB sequences of human procathepsin B; File S2: Sequence alignment of the UniProt and PDB sequences of human procathepsin H; File S3: Sequence alignment of the UniProt and PDB sequences of human procathepsin X; File S4: Sequence alignment of the UniProt and PDB sequences of human procathepsin C; Figure S1: Schematic representation of the multiple sequence alignment of human procathepsins B, C, H and X.

## Abbreviations

6-OHDA	6-hydroxydopamine
α-Syn	α-Synuclein
AD	Alzheimer’s disease
ALS	Amyotrophic Lateral Sclerosis
CATH	Class (C), Architecture (A), Topology (T), and Homologous superfamily (H)
CNS	Central Nervous System
EAE	Experimental Autoimmune Encephalomyelitis
ECM	Extracellular matrix
GBA	Glucosylceramidase β1
GCase	Glucocerebrosidase
HD	Huntington’s disease
IFN-β	Interferon-β
IκBα	κBα inhibitor
LRRK2	Leucine-rich repeat serine/threonine-protein kinase 2
MS	Multiple sclerosis
NF-κB	Nuclear factor-κB
PD	Parkinson’s disease
PDB	Protein Data Bank
ROS	Reactive Oxygen Species
SOD1	Superoxide dismutase [Cu-Zn]
Tg	Transgenic mouse
TLR	Toll-like receptor
TME	Tumour microenvironment
TNFα	Tumour necrosis factor α
UniProt	Universal Protein Knowledgebase
