# Supplementary material for: The Role of Cysteine Protease Cathepsins B, H, C, and X/Z in Neurodegenerative Diseases and Cancer
Source: Int J Mol Sci. 2023 Oct 26;24(21):15613. doi: 10.3390/ijms242115613 (PMC10650516; doi:10.3390/ijms242115613)
Supplement: Supplementary file 1 [file ijms-24-15613-s001.zip › Supplementary Materials-final.pdf]

Supplementary Materials

Table S1. Classification of human cathepsins according to the MEROPS database of proteolytic enzymes. The data was retrieved from the MEROPS database [39].

File S1. Sequence alignment of the UniProt and PDB sequences of human procathepsin B. The sequence alignment between human procathepsin B [76] 1D (UniProtKB ID P07858 [52] (upper panel) and 3D (PDB ID: 3pbh:A, [77]) (lower panel) was retrieved from the PDBSum web server [78]. The differences between the PDB and UniProt sequences are indicated with a black cross (X).

File S2. Sequence alignment of the UniProt and PDB sequences of human procathepsin H. The sequence alignment between human procathepsin H [79] 1D (UniProtKB ID P09668 [52] (upper panel) and 3D (PDB ID: 6czk:A, [60]) (lower panel) was retrieved from the PDBSum web server [78]. An amino residue difference between the PDB and UniProt sequences is indicated with a black cross (X).

File S3. Sequence alignment of the UniProt and PDB sequences of human procathepsin X. **(A)** The sequence alignment between human procathepsin X [50, 51] 1D (UniProtKB ID Q9UBR2 [52] (upper panel) and 3D (PDB ID: 1deu:A, [75]) (lower panel) was retrieved from the PDBSum web server [78]. An amino residue difference between the PDB and UniProt sequences is indicated with a black cross (X). **(B)** The sequence alignment between human procathepsin X [50, 51] 1D (UniProtKB ID Q9UBR2 [52] (upper panel) and 3D (PDB ID: 1deu:B, [75]) (lower panel) was retrieved from the PDBSum web server [78]. The differences between the PDB and UniProt sequences are indicated with a black cross (X).

File S4. Sequence alignment of the UniProt and PDB sequences of human procathepsin C. The sequence alignment between human procathepsin C [80] 1D (UniProtKB ID P53634 [52] (upper panel) and 3D (PDB ID: 3pdf:A, [81]) (lower panel) was retrieved from the PDBSum web server [78]. The differences between the PDB and UniProt sequences are indicated with a black cross (X).

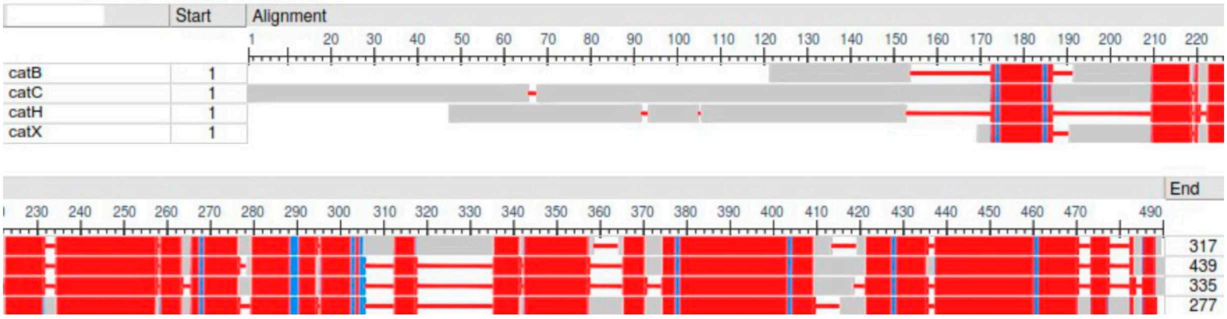

Figure S1. Schematic representation of the multiple sequence alignment of human procathepsins B, C, H, and X. The multiple sequence alignment of human procathepsin B (catB; 1pbh:A [82]), human 8 procathepsin C (catC; P53634|25-463; [80]), human procathepsin H (catH; 6czk:A; [60]), and human procathepsin X (catX; 1deu:A; [75]) was performed using a constrained-based multiple alignment tool (COBALT) [Papadopoulos, J. S.; Agarwala, R., *Bioinformatics*. **2007**, *23*, (9), 1073-9].

References

39. Rawlings, N. D. ; Barrett, A. J.; Thomas, P. D. ; Huang, X.; Bateman, A.; Finn, R. D., The MEROPS database of proteolytic enzymes, their substrates and inhibitors in 2017 and a comparison with peptidases in the PANTHER database. *Nucleic Acids Res*. **2018**, *46*(D1), D624-D632.

50. Nägler, D. K.; Ménard, R., Human cathepsin X: a novel cysteine protease of the papain family with a very short proregion and unique insertions. *FEBS Lett.* **1998,** *434*, (1-2), 135-9.
51. Santamaría, I.; Velasco, G.; Pendás, A. M.; Fueyo, A.; López-Otín, C., Cathepsin Z, a novel human cysteine proteinase with a short propeptide domain and a unique chromosomal location. *J. Biol. Chem.* **1998,** *273*, (27), 16816-23.
52. UniProt Consortium, UniProt: the Universal Protein Knowledgebase in 2023. *Nucleic Acids Res.* **2023**, *51*, (D1), D523-D31.
60. Hao, Y.; Purtha, W.; Cortesio, C.; Rui, H.; Gu, Y.; Chen, H.; Sickmier, E. A.; Manzanillo, P.; Huang, X., Crystal structures of human procathepsin H. *PLoS One* **2018,** *13*, (7), e0200374.
75. Sivaraman, J.; Nägler, D. K.; Zhang, R.; Ménard, R.; Cygler, M., Crystal structure of human procathepsin X: a cysteine protease with the proregion covalently linked to the active site cysteine. *J. Mol. Biol.* **2000,** *295*, (4), 939-51.
76. Chan, S. J.; San Segundo, B.; McCormick, M. B.; Steiner, D. F., Nucleotide and predicted amino acid sequences of cloned human and mouse preprocathepsin B cDNAs. *Proc. Natl. Acad. Sci. U. S. A.* **1986,** *83*, (20), 7721-5.
77. Podobnik, M.; Kuhelj, R.; Turk, V.; Turk, D., Crystal structure of the wild-type human procathepsin B at 2.5 A resolution reveals the native active site of a papain-like cysteine protease zymogen. *J. Mol. Biol.* **1997,** *271*, (5), 774-88.
78. Laskowski, R. A; Jabłońska, J.; Pravda, L.; Vařeková, R. S.; Thornton, J. M., PDBsum: Structural summaries of PDB entries. *Protein Sci.* **2018**, *27*, (1), 129-134.
79. Fuchs, R.; Gassen, H. G., Nucleotide sequence of human preprocathepsin H, a lysosomal cysteine proteinase. *Nucleic Acids Res.* **1989,** *17*, (22), 9471.
80. Paris, A.; Strukelj, B.; Pungercar, J.; Renko, M.; Dolenc, I.; Turk, V., Molecular cloning and sequence analysis of human preprocathepsin C. *FEBS Lett.* **1995,** *369*, (2-3), 326-30.
81. Lainé, D.; Palovich, M.; McCleland, B.; Petitjean, E.; Delhom, I.; Xie, H.; Deng, J.; Lin, G.; Davis, R.; Jolit, A.; Nevins, N.; Zhao, B.; Villa, J.; Schneck, J.; McDevitt, P.; Midgett, R.; Kmett, C.; Umbrecht, S.; Peck, B.; Davis, A. B.; Bettoun, D., Discovery of novel cyanamide-based inhibitors of cathepsin C. *ACS Med Chem Lett.* **2010**, *2*, (2), 142-7.
82. Turk, D.; Podobnik, M.; Kuhelj, R.; Dolinar, M.; Turk, V., Crystal structures of human procathepsin B at 3.2 and 3.3 Angstroms resolution reveal an interaction motif between a papain-like cysteine protease and its propeptide. *FEBS Lett.* **1996,** *384*, (3), 211-4.
